# Glucose-insulin-potassium improves left ventricular performances after aortic valve replacement: a secondary analysis of a randomized controlled trial

**DOI:** 10.1186/s12871-019-0845-0

**Published:** 2019-09-06

**Authors:** Marc Licker, John Diaper, Tornike Sologashvili, Christoph Ellenberger

**Affiliations:** 1 Department of Anesthesiology, Pharmacology and Intensive Care, University Hospital of Geneva, CH-1211 Geneva, Switzerland; 2 Faculty of Medicine, University of Geneva, CH-1211 Geneva, Switzerland; 3 Division of Cardiovascular Surgery, University Hospital of Geneva, Geneva, Switzerland; 4 Department of Anesthesiology, Pharmacology and Intensive Care, University Hospital Geneva & Faculty of Medicine, CH-1206 Geneva, Switzerland

**Keywords:** Aortic valve stenosis, Echocardiography, Myocardial protection

## Abstract

**Background:**

Patients with left ventricular (LV) hypertrophy may suffer ischemia-reperfusion injuries at the time of cardiac surgery with impairment in left ventricular function. Using transesophageal echocardiography (TEE), we evaluated the impact of glucose-insulin potassium (GIK) on LV performances in patients undergoing valve replacement for aortic stenosis.

**Methods:**

In this secondary analysis of a double-blind randomized trial, moderate-to-high risk patients were assigned to receive GIK (20 IU insulin with 10 mEq KCL in 50 ml glucose 40%) or saline over 60 min upon anesthetic induction. The primary outcomes were the early changes in 2-and 3-dimensional left ventricular ejection fraction (2D and 3D-LVEF), peak global longitudinal strain (PGLS) and transmitral flow propagation velocity (Vp).

**Results:**

At the end of GIK infusion, LV-FAC and 2D- and 3D-LVEF were unchanged whereas Vp (mean difference [MD + 7.9%, 95% confidence interval [CI] 3.2 to 12.5%; *P* <  0.001) increased compared with baseline values. After Placebo infusion, there was a decrease in LV-FAC (MD -2.9%, 95%CI − 4.8 to − 1.0%), 2D-LVEF (MD -2.0%, 95%CI − 2.8 to − 1.3%, 3D-LVEF (MD -3.0%, 95%CI − 4.0 to − 2.0%) and Vp (MD − 4.5 cm/s, 95%CI − 5.6 to − 3.3 cm/s).

After cardiopulmonary bypass, GIK pretreatment was associated with preserved 2D and 3D-LVEF (+ 0.4%, 95% 95%CI − 0.8 to 1.7% and + 0.4%, 95%CI − 1.3 to 2.0%), and PGLS (− 0.9, 95%CI − 1.6 to − 0.2) as well as higher Vp (+ 5.1 cm/s, 95%CI 2.9 to 7.3), compared with baseline. In contrast, in the Placebo group, 2D-LVEF (− 2.2%, 95%CI − 3.4 to − 1.0), 3D-LVEF (− 6.0%, 95%CI − 7.8 to − 4.2), and Vp (− 7.6 cm/s, 95%CI − 9.4 to − 5.9), all decreased after bypass.

**Conclusions:**

Administration of GIK before aortic cross-clamping resulted in better preservation of systolic and diastolic ventricular function in patients with LV hypertrophy undergoing aortic valve replacement.

**Trial registration:**

ClinicalTrials.gov: NCT00788242, registered on November 10, 2008.

## Introduction

Currently, aortic valve replacement (AVR) remains the standard of care to treat patients with severe aortic valvular stenosis, although elderly and high-risk patients may now benefit from a lesser invasive transarterial vascular approach [1]. Low cardiac output syndrome occurs in 5 to 15% of patients undergoing open heart surgery and is a main cause of mortality [2]. Following AVR, patients with aortic stenosis are prone to develop myocardial injuries and contractile dysfunction owing to difficulties in protecting the hypertrophic heart with cardioplegic solutions [2, 3].

The term “postcardiotomy ventricular dysfunction” (PCVD) has been coined to define new onset or worsening heart failure that develops following weaning from cardiopulmonary bypass (CPB) and that requires support with inotropes [4]. Transesophageal echocardiography (TEE) coupled with haemodynamic monitoring allows the cardiac team to distinguish PCVD from other functional or structural abnormalities such as valve prosthesis/patient mismatch, myocardial ischemia or systolic anterior motion of the anterior mitral leaflet [5, 6].

In animal models of ischemia-reperfusion, there is strong evidence that the infusion of glucose-insulin-potassium (GIK) minimizes myocardial injuries [7, 8]. In patients undergoing open heart surgery, although the administration of GIK has been shown to improve cardiac output, few and conflicting results have been reported regarding functional ventricular performances [9, 10].

The aim of this study was to investigate the changes in left ventricular function using TEE, in moderate-to-high risk patients undergoing AVR.

## Materials and methods

With ethical approval from the local ethics commission (CER 08–095), a randomized controlled blinded trial was conducted at the University Hospital of Geneva and was registered November 10, 2008 on ClinicalTrials.gov (NCT00788242). Written consent was obtained from each eligible participant. The trial was conducted in accordance with the Consolidated Standards of Reporting Trials (CONSORT) 2010 statement [11].

From January 1, 2009 to December 31, 2013, adult patients with severe aortic valve stenosis and/or coronary artery disease scheduled for elective AVR and/or coronary artery bypass surgery (CABGS) were enrolled if they had a Parsonnet score higher than 7. Exclusion criteria were the presence of poorly controlled diabetes mellitus, liver disease (Child-Pugh C stage), dementia, cerebrovascular disease or contraindications for TEE.

Results regarding clinical outcomes and the incidence of PCVD (main study endpoints) in the whole population have been reported previously as well as the effects of GIK on TEE parameters (secondary endpoints) in the CABGS subpopulation [12, 13]. In the current report and as preplanned, we analyzed the effects of GIK infusion on TEE parameters before and after CPB in patients who underwent isolated AVR (without CABGS), in whom TEE was completed with good quality imaging.

**Fig. 1:**
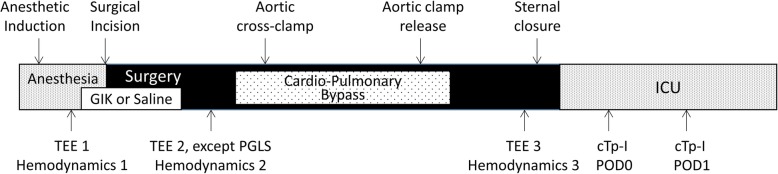
Time line of study protocol describing the study interventions (saline vs glucose-insuline-potassium), surgical/anesthetic events and data collection

The randomization and blinding process as well as perioperative care has been described elsewhere in detail [12]. In short, patients were randomized in two groups (1:1), receiving an unlabeled coded solution (NaCl 0.9%, in Placebo group or Actrapid, Novo Nordisk 20 IU and potassium chloride 10 mEq in 50 ml of 40% glucose, in GIK group) over 60 min upon anesthetic induction (Fig. 1). A standard anesthesia technique was applied that included inhaled sevoflurane for myocardial preconditioning and intrathecal morphine analgesia to minimize the administration of opiates and facilitate early extubation. All surgical procedures were performed via sternotomy, under normothermic nonpulsatile CPB. Weaning from CPB was standardized and guided by TEE and hemodynamic measurements [14].

The primary outcome variable was the left ventricular ejection fraction (LVEF) as measured by two- and three dimensional (2D and 3D) echocardiography, peak global longitudinal strain (PGLS) and transmitral flow propagation velocity (Vp). Secondary study endpoints included other TEE parameters as well as hemodynamic parameters. TEE data acquisition was performed intraoperatively by two experienced echocardiographers at three time points, before drug infusion, 20 min after drug infusion and at the end of surgery (Fig. 1) using an iE33 ultrasound system (Philips Medical System, Einthoven, Netherland). The acquisition process has previously been described in detail [13]. In short, a comprehensive TEE examination was performed. 2D-LVEF was assessed using the Simpson’s method of discs. 3D-LVEF was assessed from a full volume scan of the left ventricle (with 4 R-wave triggered sub-volumes) using the QLAB 3D-advanced quantification software package. Speckle-tracking analysis to assess PGLS was performed with the cardiac motion quantification software (CMQ-Advanced; Philips Healthcare, Einthoven, Netherland). Transmitral flow propagation velocity (Vp) was determined from the mid-esophageal 4-chamber view using the color M-mode. Intraobserver and interobserver variabilities for 2-D/3D LVEF, PGLS and Vp were studied off-line in randomly selected patients (*n* = 10).

Details on the statistical analysis have been given previously [13]. Summary descriptive statistics are expressed as frequencies (and percentages, %), medians (and interquartile range, IQR 25–75%), and means (and standard deviations, SD). Two-sided unpaired t tests, Wilcoxon rank-sum tests, chi-squared tests, and repeated-measures two-way analysis of variance (ANOVA) was used to estimate between and within group differences when appropriate. Inter- and intra-observer variabilities in echocardiographic measurements were assessed using the Pearson’s correlation coefficient. Statistical tests were conducted using STATA 14 software (Stata Corp, College Station, TX, USA).

## Results

**Fig. 2:**
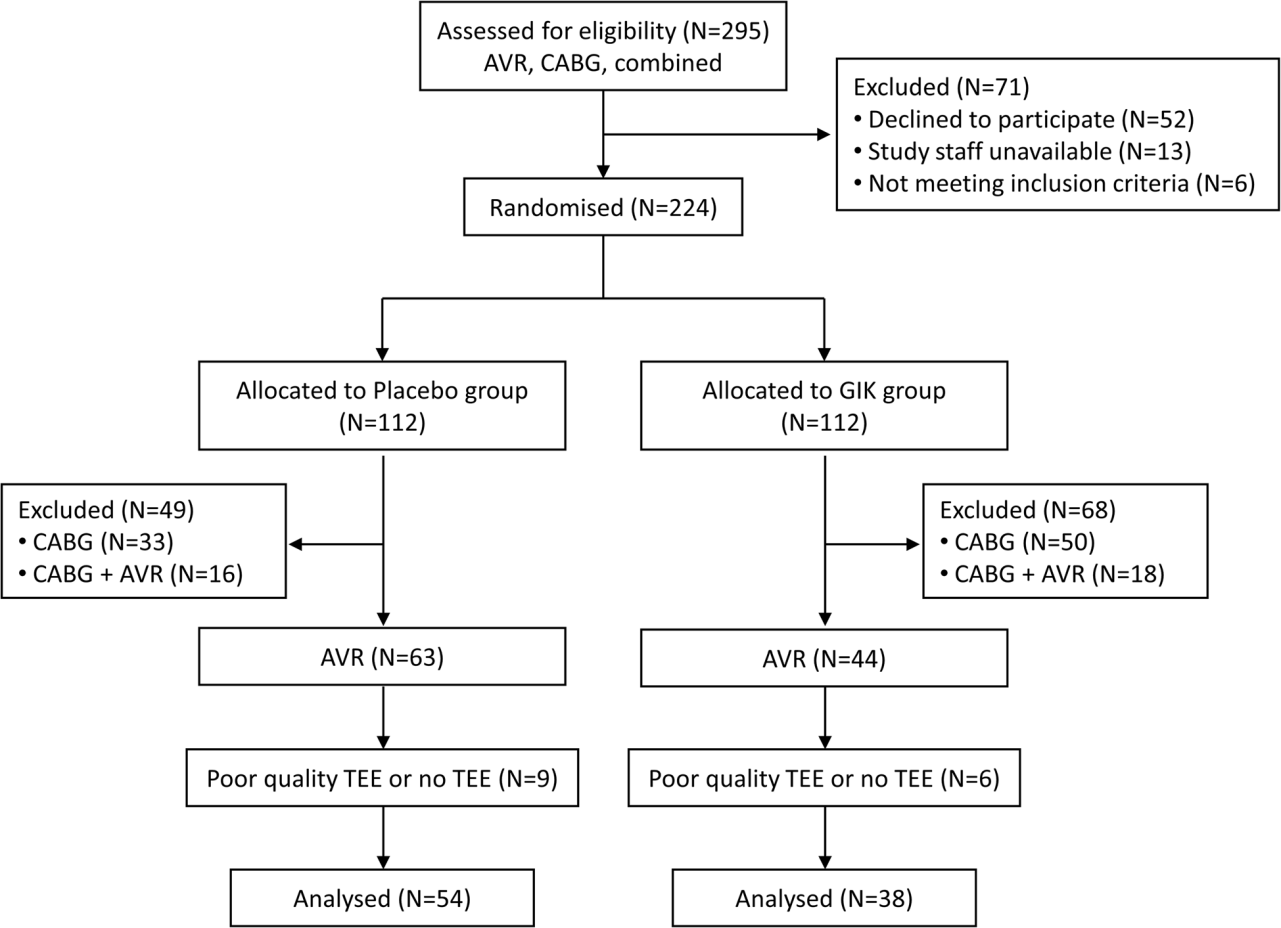
Consolidated Standards of Reporting Trials flow diagram. AVR, aortic valve replacement; CABGS, coronary artery bypass graft surgery; GIK, glucose-insuline-potassium; TEE, transesophageal echocardiography

The Consolidated Standards of Reporting Trials (CONSORT) diagram is shown in Fig. 2. From a total of 295 screened patients, 212 were randomized into GIK and Placebo groups (110 and 112, respectively). Among those undergoing isolated AVR, 63 and 44 were allocated to Placebo and GIK groups, respectively. After exclusion of cases with unavailable or poor quality TEE (*N* = 15), 92 patients remained for final analysis (Placebo, *N* = 54 and GIK, *N* = 38).

As shown in Tables 1, the two groups were well balanced in baseline preoperative variables and surgical characteristics. Intraoperatively, BGC were similar in the two groups, with no difference regarding the need for glucose infusion (GIK, 4 (7%) vs Placebo 3 (4%), respectively, *P* = 0.689) and insulin being added more frequently in the GIK group (24 (44%) vs 14 (20%) in Placebo, *P* = 0.004). Strong intra-rater and inter-rater reproducibility for all TEE parameters was reported as correlation coefficients with 95%CI (Table 2).

**Table 1:** Clinical and surgical characteristics of patients undergoing aortic valve replacement and receiving Saline or Glucose-Insulin Potassium (GIK) infusion

Characteristics	Placebo		GIK		*P* value
	(N = 54)		(N = 38)		
*Demographics*					
Age, years^a^	73.2	(9.6)	71.7	(9.8)	0.464^b^
Body Mass index^a^	29.5	(6.1)	27.7	(4.5)	0.128^b^
Sex, male	33	(61.1)	20	(52.6)	0.418
*Comorbidities*					
Bernstein-Parsonnet score^a^	21.8	(7.5)	20.8	(8.3)	0.547^b^
Hypertension	47	(87.0)	37	(97.4)	0.083
Pulmonary Hypertension	2	(3.7)	3	(7.9)	0.645^c^
Hypercholesterolemia	37	(68.5)	29	(76.3)	0.413
Diabetes mellitus	17	(31.5)	13	(34.2)	0.783
Vascular disease	23	(42.6)	15	(39.5)	0.765
Chronic Obstructive Lung Disease	6	(11.1)	2	(5.3)	0.463^c^
Previous cardiac surgery	3	(5.6)	1	(2.6)	0.640^c^
*Preoperative blood parameters*					
Hemoglobin, g/dL^a^	12.5	(2.1)	12.4	(2.0)	0.747^b^
Creatinine clearance, ml/min ^a^	81.1	(34.6)	75.4	(30.0)	0.418^b^
*Surgical data*					
CPB time, min^a^	97.1	(37.5)	102.2	(47.8)	0.564^b^
Aortic clamping time, min^a^	74.3	(29.0)	76.5	(32.0)	0.730^b^
*Intraoperative fluids and blood*					
Crystalloids and colloids, ml^a^	3′213	(1214)	2′897	(850)	0.170^b^
Blood transfusion	31	(57.4)	26	(68.4)	0.284
Fresh frozen plasma	12	(22.2)	9	(23.7)	0.869
Platelets	8	(14.8)	4	(10.5)	0.362
Blood glucose (mMol/L)					
Start of surgery^a^	6.7	(1.5)	6.7	(1.6)	0.980^b^
Before bypass^a^	7.4	(1.6)	7.6	(2.9)	0.621^b^
During Bypass^a^	7.4	(1.7)	7.1	(2.5)	0.556^b^
End of surgery^a^	7.6	(1.9)	6.8	(2.1)	0.158^b^

Data given as number (percentage) unless otherwise indicated. Chi-squared tests were used for statistical tests unless otherwise indicated. ^a^ Data given as mean (standard deviation); ^b^ student t test. ^c^ Fisher exact test

*AVR* aortic valve replacement, *CABG* coronary artery bypass grafting, *CPB* cardiopulmonary bypass

**Table 2:** Interobserver and intraobserver variability for measurements of transesophageal echocardiographic data

Measurements	Interobserver Correlation Coefficient	95% Confidence Interval	Intraobserver Correlation Coefficient	95% Confidence Interval
Vp	0.742	0.488–0.945	0.791	0.477–0.944
FAC	0.956	0.890–0.983	0.883	0.723–0.953
2D-LVEF	0.890	0.739–0.956	0.923	0.812–0.970
3D-LVEF	0.819	0.591–0.926	0.840	0.595–0.973
PGLS	0.856	0.571–0.908	0.899	0.671–0.943

Vp, transmitral flow propagation velocity; FAC, fractional area change; 2D-LVEF-, two-dimensional left ventricular ejection fraction; 3D-LVEF-, three-dimensional left ventricular ejection fraction; PGLS, peak global longitudinal strain

At baseline, patients presented similarly increased LV posterior wall thickness (1.19 ± 0.23 mm and 1.21 ± 0.19 mm in Placebo and GIK groups, respectively; *P* = 0.543) whereas LV-FAC, 2DLVEF, 3D-LVEF and Vp were lower in the GIK group compared with the Placebo group (Table 3).

**Table 3:** Echocardiographic parameters in patients undergoing aortic valve replacement and receiving Placebo or Glucose-Insulin Potassium (GIK) infusion

Parameter	Start surgery		After GIK		End Surgery		*P*-value
Preload							
End diastolic area (cm^2^)							
All patients	13.9	(3.3)	13.2	(3.0)	12.6	(3.4)	< 0.001
Placebo group	13.6	(2.7)	12.9	(2.4)	12.3	(2.9)	< 0.001
GIK group	14.3	(4.5)	13.5	(3.8)	13.0	(4.1)	0.001
				*Baseline difference*			*0.362*
				*Effect modification by GIK*			*0.949*
*Systolic function*							
LV FAC (%)							
All patients	47.1	(6.2)	45.4	(8.4)	44.7	(7.8)	0.033
Placebo group	48.4	(6.1)	45.5	(8.5)	42.7	(8.5)	< 0.001
GIK group	45.2	(6.0)	45.2	(8.4)	47.5	(5.8)	0.052
				*Baseline difference*			*0.016*
				*Effect modification by GIK*			*< 0.001*
3D-LVEF (%)							
All patients	47.5	(6.4)	46.2	(5.5)	44.1	(6.4)	< 0.001
Placebo group	49.3	(5.4)	46.3	(5.2)	43.3	(6.8)	< 0.001
GIK group	44.9	(6.9)	46.0	(5.9)	45.2	(5.7)	0.236
				*Baseline difference*			*< 0.001*
				*Effect modification by GIK*			*< 0.001*
2D-LVEF (%)							
All patients	43.7	(5.3)	42.5	(5.4)	42.5	(5.9)	0.006
Placebo group	44.7	(4.5)	42.7	(5.3)	42.5	(6.5)	< 0.001
GIK group	42.2	(6.1)	42.4	(5.7)	42.6	(5.0)	0.722
				*Baseline difference*			*0.023*
				*Effect modification by GIK*			*0.002*
PGLS (%)							
All patients	−12.3	(2.5)	–	–	−12.6	(2.1)	0.151
Placebo group	−12.6	(2.3)	–	–	−12.6	(1.9)	0.985
GIK group	−11.8	(2.7)	–	–	−12.6	(2.4)	0.014
				*Baseline difference*			*0.157*
				*Effect modification by GIK*			*0.076*
LV systolic strain rate (s^−1^)							
All patients	−1.04	(0.29)	–	–	−1.07	(0.24)	0.174
Placebo group	−1.07	(0.26)	–	–	−1.07	(0.25)	1.000
GIK group	−0.99	(0.32)	–	–	−1.07	(0.23)	0.053
				*Baseline difference*			*0.202*
				*Effect modification by GIK*			*0.094*
*Diastolic function*							
E-wave velocity (cm/s)							
All patients	58.8	(14.2)	56.9	(13.6)	58.2	(16.3)	0.505
Placebo group	59.8	(13.5)	56.3	(13.4)	56.2	(17.8)	0.229
GIK group	57.2	(15.3)	57.7	(14.0)	61.0	(13.7)	0.286
				*Baseline difference*			*0.391*
				*Effect modification by GIK*			*0.136*
A-wave velocity (cm/s)							
All patients	59.5	(16.0)	58.4	(15.4)	58.8	(19.2)	0.707
Placebo group	57.9	(14.3)	58.0	(14.1)	63.1	(20.6)	0.047
GIK group	61.8	(18.0)	58.9	(17.3)	52.8	(15.2)	< 0.001
				*Baseline difference*			*0.249*
				*Effect modification by GIK*			*< 0.001*
E/A ratio							
All patients	1.06	(0.43)	1.05	(0.41)	1.09	(0.54)	0.660
Placebo group	1.12	(0.48)	1.03	(0.38)	0.99	(0.62)	0.274
GIK group	0.99	(0.34)	1.08	(0.46)	1.22	(0.39)	< 0.001
				*Baseline difference*			*0.161*
				*Effect modification by GIK*			*0.007*
Pressure half-time (ms)							
All patients	55.0	(14.9)	53.8	(13.7)	51.3	(13.9)	0.210
Placebo group	54.6	(14.3)	54.4	(14.4)	51.0	(13.4)	0.851
GIK group	55.6	(15.9)	52.9	(12.8)	51.7	(14.8)	0.082
				*Baseline difference*			*0.757*
				*Effect modification by GIK*			*0.235*
Isovolemic relaxation time (ms)							
All patients	88.3	(37.0)	89.0	(35.7)	83.6	(33.5)	0.133
Placebo group	90.8	(37.9)	87.4	(36.2)	84.5	(37.6)	0.229
GIK group	84.7	(35.7)	91.2	(35.4)	82.4	(27.0)	0.126
				*Baseline difference*			*0.440*
				*Effect modification by GIK*			*0.213*
S-wave velocity (LUPV) (cm/s)							
All patients	30.8	(9.3)	29.7	(9.7)	27.6	(9.5)	0.015
Placebo group	31.7	(9.6)	29.4	(9.8)	26.7	(9.5)	0.011
GIK group	29.4	(8.8)	30.2	(9.5)	28.9	(9.5)	0.336
				*Baseline difference*			*0.247*
				*Effect modification by GIK*			*0.015*
D-wave velocity (LUPV) (cm/s)							
All patients	23.2	(6.8)	22.1	(6.7)	22.3	(10.5	0.397
Placebo group	22.8	(6.9)	22.8	(7.6)	23.8	(11.8)	0.652
GIK group	23.8	(6.8)	21.2	(4.9)	20.3	(6.7)	0.005
				*Baseline difference*			*0.520*
				*Effect modification by GIK*			*0.065*
A-wave velocity (LUPV) (cm/s)							
All patients	11.8	(4.2)	11.8	(4.3)	9.8	(4.8)	< 0.001
Placebo group	12.4	(4.2)	12.1	(4.8)	7.9	(4.4)	< 0.001
GIK group	10.8	(4.0)	11.3	(3.6)	12.4	(4.1)	0.076
				*Baseline difference*			*0.066*
				*Effect modification by GIK*			*< 0.001*
S/D ratio							
All patients	1.42	(0.59)	1.44	(0.62)	1.42	(0.68)	0.871
Placebo group	1.46	(0.49)	1.38	(0.53)	1.35	(0.69)	0.429
GIK group	1.35	(0.71)	1.52	(0.73)	1.52	(0.66)	0.119
				*Baseline difference*			*0.385*
				*Effect modification by GIK*			*0.080*
Early lateral velocity (cm/s)							
All patients	10.2	(2.9)	9.5	(2.7)	8.2	(2.3)	< 0.001
Placebo group	10.4	(2.9)	9.5	(2.7)	7.4	(2.2)	< 0.001
GIK group	9.8	(2.9)	9.6	(2.7)	9.3	(2.1)	0.274
				*Baseline difference*			*0.387*
				*Effect modification by GIK*			*< 0.001*
Late lateral velocity (cm/s)							
All patients	9.0	(2.5)	8.7	(2.2)	7.5	(2.2)	< 0.001
Placebo group	9.0	(2.7)	8.9	(2.2)	7.8	(2.4)	0.002
GIK group	8.9	(2.2)	8.4	(2.2)	6.9	(1.7)	< 0.001
				*Baseline difference*			*0.815*
				*Effect modification by GIK*			*0.236*
Early septal velocity (cm/s)							
All patients	6.1	(1.6)	5.7	(1.5)	4.9	(1.4)	< 0.001
Placebo group	6.0	(1.5)	5.6	(1.5)	4.5	(1.2)	< 0.001
GIK group	6.3	(1.6)	5.9	(1.6)	5.6	(1.4)	0.010
				*Baseline difference*			*0.445*
				*Effect modification by GIK*			*0.019*
Late septal velocity (cm/s)							
All patients	5.8	(1.9)	5.5	(1.8)	4.5	(1.7)	< 0.001
Placebo group	5.6	(2.0)	5.3	(1.9)	4.8	(1.8)	0.003
GIK group	6.0	(1.8)	5.7	(1.6)	4.1	(1.4)	< 0.001
				*Baseline difference*			*0.342*
				*Effect modification by GIK*			*0.003*
E/e’ ratio							
All patients	6.2	(2.4)	6.4	(2.5)	7.6	(3.1)	< 0.001
Placebo group	6.2	(2.2)	6.4	(2.8)	8.2	(3.7)	< 0.001
GIK group	6.3	(2.6)	6.4	(2.0)	6.8	(1.7)	0.342
				*Baseline difference*			*0.828*
				*Effect modification by GIK*			*0.026*
Flow Propagation Velocity (cm/s)							
All patients	42.6	(7.3)	41.3	(6.7)	40.2	(7.0)	0.014
Placebo group	44.0	(6.9)	39.5	(5.9)	36.3	(6.1)	< 0.001
GIK group	40.6	(7.6)	43.8	(7.0)	45.7	(3.9)	< 0.001
				*Baseline difference*			*0.030*
				*Effect modification by GIK*			*< 0.001*

Data given as mean (standard deviation)

Repeated-measures two-way analysis of variance (ANOVA) with Greenhouse-Geisser correction was used to estimate trend differences between and within group differences

LV FAC, left ventricular fractional area change; 3D-LVEF, three-dimensional left ventricular ejection fraction; 2D-LVEF, two-dimensional left ventricular ejection fraction; PGLS, peak global longitudinal strain; LUPV, left upper pulmonary vein

Throughout the three study periods, GIK infusion produced strong interaction effects on LVFAC, 2D-LVEF, 3D-LVEF and Vp (*p* <  0.001). At the end of GIK infusion, LV-FAC and 2D- and 3D-LVEF were unchanged whereas Vp (mean difference [MD + 7.9%, 95% confidence interval [CI] 3.2 to 12.5%; *P* <  0.001) increased compared with baseline values (Table 3). After Placebo infusion, we observed decreases in LV-FAC (MD -2.9%, 95%CI − 4.8 to − 1.0%), 2D-LVEF (MD -2.0%, 95%CI − 2.8 to − 1.3%, 3D-LVEF (MD -3.0%, 95%CI − 4.0 to − 2.0%) and Vp (MD − 4.5 cm/s, 95%CI − 5.6 to − 3.3 cm/s) compared with baseline values.

After separation from CPB, mean transprosthetic pressure gradients were comparable in the two groups (6 mmHg [2] in Placebo and 7 mmHg [2] in GIK, *P* = 0.463).

**Fig. 3:**
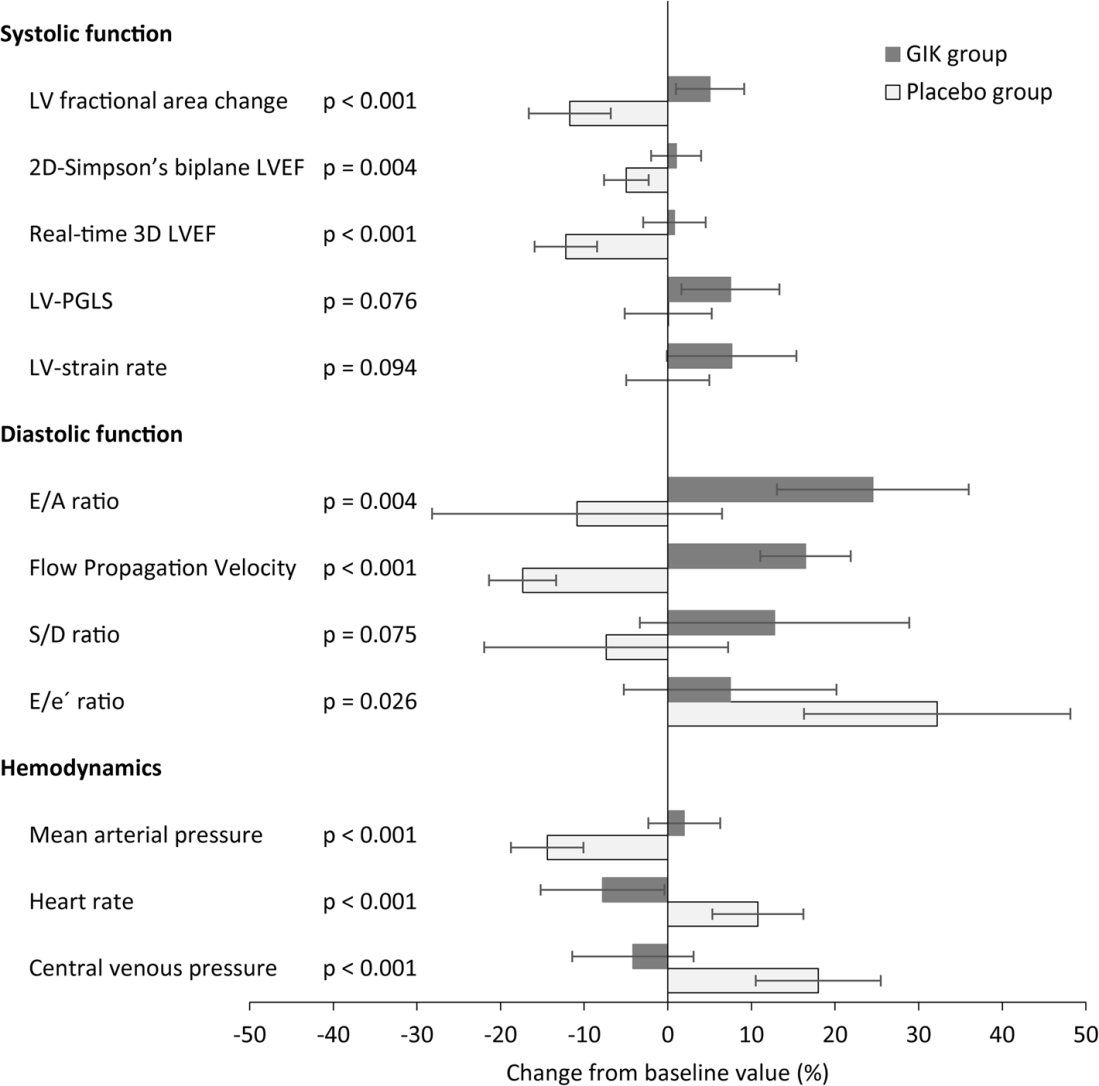
Hemodynamic and echocardiographic changes from baseline after study drug administration and at the end of surgery in patients undergoing aortic valve replacement and receiving Placebo or Glucose-Insulin Potassium (GIK) infusion

Compared with baseline values, LVFAC, 2D-LVEF and 3D-LVEF, all decreased at the end of surgery in the Placebo group, [MD] -5.7%, *P* <  0.001; MD -2.2%, P <  0.001; MD -6.0%, P <  0.001, respectively) whereas these indices of systolic LV function improved or remained unchanged in the GIK group (MD + 2.3%; *P* = 0.017, MD, + 0.4%, *P* = 0.503, MD + 0.4%, *P* = 0.671, respectively) (Fig. 3). Patients receiving GIK presented minor changes in PGLS (MD -0.9, *P* = 0.014) and LV strain rate (MD -0.08, *P* = 0.053). In the Placebo group, there was no change in PGLS and LV strain rate from pre-bypass to post-bypass condition.

In the GIK group, the E/A ratio and Vp were higher at the end of surgery compared with baseline (MD + 19.5%, *P* < 0.001; MD + 5.1 cm/s, *P* < 0.001, respectively) and compared with the Placebo group. As indicators of cardiac preload, the E/e’ ratio was increased at the end of surgery, compared with baseline, in the Placebo group (MD 32.2%, 95%CI 16.3 to 48.1%, *P* < 0.001) whereas this cardiac filling parameter remained unchanged in the GIK group.

After weaning from CPB, GIK pretreated patients less frequently required norepinephrine (11 [29.0%] vs 44 (81.5%], in the Placebo group), dobutamine (5 [13.2%] vs 29 [53.7%] in the Placebo group), epinephrine (1 [2.6%] vs 7 (13.0%], in the Placebo group), or a combination of at least two inotropes (4 [10.5%] vs 32 [59.3%] in the Placebo group).

## Discussion

In this randomized controlled trial including patients undergoing isolated AVR for aortic stenosis, we demonstrated that the infusion of GIK, − in addition to usual cardioprotective techniques -, prevented the early impairment in LV systolic and diastolic function following separation from CPB and resulted in lesser requirement of cardiovascular drug support. The extent of the benefit was similar to that seen in patients undergoing CABGS [13].

Patients included in this trial are likely to correspond to recent evolution of real-world cardiac surgery. Using the Parsonnet score, the increased operative risk profile was mainly related to hypertension (91% of patients), advanced age (61% ≥70 years) hyperlipidemia (72%) and diabetes mellitus (33%), all factors known to be implicated in promoting LV hypertrophy and impaired LV function. The development of valvular aortic stenosis was another trigger for structural remodeling of the LV as characterized by cardiomyocytes hypertrophy and apoptosis, decreased coronary flow reserve, reduced capillary density, as well as intercellular matrix fibrosis [3, 15]. In the hypertrophied LV, the relative deficient microcirculation hinders the delivery of the cardioplegic solution particularly to the subendocardium, therefore compromising intra-operative myocardial preservation and rendering the heart more susceptible to ischemia-reperfusion injuries following weaning from CPB as manifested by early deterioration of LV performances and release of myocardial biomarkers [3, 5, 15].

In both groups, standardized cardioprotective strategies were applied including antegrade administration of cold blood cardioplegia and pre-ischemic exposure to volatile anesthetics. Although no clear benefit has so far been demonstrated by varying the composition of cardioplegia or its delivery (retrograde vs antegrade), many cardiac teams have adopted the infusion of cold oxygenated blood as it provides effective buffering and uniform capillary flow through the myocardium [16–18]. Anesthetic preconditioning may also enhance cardioprotection by modulating mitochondrial electron pathways and ATP level through protein kinase C and K_ATP_ channels [19, 20].

Besides standard 2D TEE examination, additional imaging techniques including 3D echocardiography and speckle tracking have been used to improve the reliability of the TEE assessment. Three dimensional echocardiography has shown an excellent agreement with magnetic resonance imaging in assessing LV function [21] whereas quantification of systolic longitudinal fiber shortening is particularly valuable in patients with LV hypertrophy since the subendocardial longitudinal fibers are more sensitive to ischemia and wall stress [22]. Abnormal patterns of deformation have been documented in the setting of preserved LVEF and changes in GLS parameters have been shown to detect early functional improvement associated with LV remodeling shortly after AVR [23].

In patients with aortic stenosis undergoing AVR, two previous randomized controlled trials have evaluated the potential cardioprotective effects of perioperative infusion of GIK and reported opposing results. Using speckle tracking echocardiography, Duncan et al. failed to demonstrate any clinically relevant improvement in longitudinal myocardial strain in patients treated by hyperinsulinemic normoglycemic clamp [24]. In contrast, in the Hypertrophy, Insulin, Glucose, and Electrolytes (HINGE) trial, Howell et al. reported a lower incidence of low cardiac output syndrome (− 70% compared with usual care group) with lesser requirement for inotropes and non-significant changes in biomarkers of myocardial injury [25]. Different patient’s populations, as well as different timing and dosing of GIK could partly explain these discrepant results. Compared with the HINGE trial, patients enrolled in Duncan’s study presented lesser degree of LV hypertrophy and well-preserved systolic LV function (mean LVEF of 62%); in addition, insulin was frequently administered in the Control group to maintain a tight glycemic control. In our trial, patients were even sicker, they had lower LVEF (mean value of 47%) compared with the HINGE trial and Duncan’s study (59% and 66%, respectively) providing more opportunity for testing cardioprotection in the intervention arm. Moreover, we limited the GIK infusion only to the pre-bypass period, in contrast with previous studies where GIK was given over the whole surgical period including bypass and post-bypass times. The hypertrophied heart is highly dependent on glucose uptake and accelerated glycolysis to fuel energy metabolism since the hypertrophied cardiomyocytes are reprogrammed with gene expression and metabolic profiles similar to the fetal hearts [26]. Under such conditions, pre-ischemic administration of GIK is expected to shift substrate utilization from fatty acids to glucose and therefore to promote more efficient oxygen utilization for synthesis of adenosine triphosphate (ATP) compounds. Besides metabolic modulation, insulin, − the key component of the GIK cocktail -, exerts other cardiovascular protective effects by improving intracellular calcium homeostasis [27] and coronary blood flow [28] as well as via phosphatidylinositol 3′-kinase-protein kinase B-endothelial nitric oxide synthase (PI3K-Akt-eNOS)-dependent signaling mechanism [8].

This study has several limitations that have already been highlighted previously [13]. Indeed, there were baseline differences in LV function between the two groups and the functional assessment was exclusively focused on the LV function. Using longitudinal strain and strain rate, various changes have been reported immediately after AVR, namely improved LV function coupled with decreased RV function that could explain the development of postoperative low cardiac output syndrome [29]. Moreover, in a similar surgical population, Maslow et al. reported that treatment with inotropes resulted in increased cardiac output that was more correlated to RV ejection fraction than to LVEF improvements [30]. Finally, we ignore whether the enhanced post-bypass LV function in GIK-treated patients may translate into better long-term clinical outcome owing to favorable LV remodeling. Repeated echocardiographic examinations over 6 to 12 months postoperative follow up period would disclose whether the GIK-related effect similar mitigates myocardial stunning or if it minimizes myocardial injuries and promotes ventricular functional recovery [31].

## Conclusions

The addition of GIK therapy to standard cardioprotective techniques in moderate-to-high risk patients with severe aortic valve stenosis, resulted in better preservation of LV systolic and diastolic function and lesser requirement of cardiovascular drug support in the early period following AVR. Further evidence is required to ascertain myocardial recovery along with improved long term survival and clinical outcome.

## Data Availability

The raw data of the current study are available from the corresponding author on request.

## Abbreviations

AVR: aortic valve replacement
BGC: blood glucose concentration
CABGS: coronary artery bypass graft surgery
CPB: cardiopulmonary bypass
GIK: glucose-insuline-potassium
LVEF: left ventricular ejection fraction
PCVD: postcardiotomy ventricular dysfunction
PGLS: peak global longitudinal strain
TEE: transesophageal echocardiography
Vp: transmitral flow propagation velocity

## References

[1] Baumgartner H, Falk V, Bax JJ, De Bonis M, Hamm C, Holm PJ, Iung B, Lancellotti P, Lansac E, Rodriguez Munoz D (2017). 2017 ESC/EACTS guidelines for the management of valvular heart disease. Eur Heart J.

[2] Lomivorotov VV, Efremov SM, Kirov MY, Fominskiy EV, Karaskov AM (2017). Low-cardiac-output syndrome after cardiac surgery. J Cardiothorac Vasc Anesth.

[3] Rader F, Sachdev E, Arsanjani R, Siegel RJ (2015). Left ventricular hypertrophy in valvular aortic stenosis: mechanisms and clinical implications. Am J Med.

[4] Mebazaa A, Pitsis AA, Rudiger A, Toller W, Longrois D, Ricksten SE, Bobek I, De Hert S, Wieselthaler G, Schirmer U (2010). Clinical review: practical recommendations on the management of perioperative heart failure in cardiac surgery. Crit Care.

[5] Barber RL, Fletcher SN (2014). A review of echocardiography in anaesthetic and peri-operative practice. Part 1: impact and utility. Anaesthesia.

[6] Licker M, Cikirikcioglu M, Inan C, Cartier V, Kalangos A, Theologou T, Cassina T, Diaper J (2010). Preoperative diastolic function predicts the onset of left ventricular dysfunction following aortic valve replacement in high-risk patients with aortic stenosis. Crit Care.

[7] LaDisa JF, Krolikowski JG, Pagel PS, Warltier DC, Kersten JR (2004). Cardioprotection by glucose-insulin-potassium: dependence on KATP channel opening and blood glucose concentration before ischemia. Am J Physiol Heart Circ Physiol.

[8] Yao H, Han X, Han X (2014). The cardioprotection of the insulin-mediated PI3K/Akt/mTOR signaling pathway. Am J Cardiovasc Drugs.

[9] Fan Y, Zhang AM, Xiao YB, Weng YG, Hetzer R (2011). Glucose-insulin-potassium therapy in adult patients undergoing cardiac surgery: a meta-analysis. Eur J Cardiothorac Surg.

[10] Bothe W, Olschewski M, Beyersdorf F, Doenst T (2004). Glucose-insulin-potassium in cardiac surgery: a meta-analysis. Ann Thorac Surg.

[11] Schulz KF, Altman DG, Moher D (2010). CONSORT 2010 statement: updated guidelines for reporting parallel group randomised trials. BMC Med.

[12] Ellenberger C, Sologashvili T, Kreienbuhl L, Cikirikcioglu M, Diaper J, Licker M (2018). Myocardial protection by glucose-insulin-potassium in moderate- to high-risk patients undergoing elective on-pump cardiac surgery: a randomized controlled trial. Anesth Analg.

[13] Licker M, Reynaud T, Garofano N, Sologashvili T, Diaper J, Ellenberger C. Pretreatment with glucose-insulin-potassium improves ventricular performances after coronary artery bypass surgery: a randomized controlled trial. J Clin Monit Comput. 2019. 10.1007/s10877-019-00280-5 PMC7223403 30788810

[14] Licker M, Diaper J, Cartier V, Ellenberger C, Cikirikcioglu M, Kalangos A, Cassina T, Bendjelid K (2012). Clinical review: management of weaning from cardiopulmonary bypass after cardiac surgery. Ann Card Anaesth.

[15] Halkos ME, Chen EP, Sarin EL, Kilgo P, Thourani VH, Lattouf OM, Vega JD, Morris CD, Vassiliades T, Cooper WA (2009). Aortic valve replacement for aortic stenosis in patients with left ventricular dysfunction. Ann Thorac Surg.

[16] Lotto AA, Ascione R, Caputo M, Bryan AJ, Angelini GD, Suleiman MS (2003). Myocardial protection with intermittent cold blood during aortic valve operation: antegrade versus retrograde delivery. Ann Thorac Surg.

[17] Ovrum E, Tangen G, Tollofsrud S, Oystese R, Ringdal MA, Istad R (2010). Cold blood versus cold crystalloid cardioplegia: a prospective randomised study of 345 aortic valve patients. Eur J Cardiothorac Surg.

[18] Braathen B, Tonnessen T (2010). Cold blood cardioplegia reduces the increase in cardiac enzyme levels compared with cold crystalloid cardioplegia in patients undergoing aortic valve replacement for isolated aortic stenosis. J Thorac Cardiovasc Surg.

[19] Jovic M, Stancic A, Nenadic D, Cekic O, Nezic D, Milojevic P, Micovic S, Buzadzic B, Korac A, Otasevic V (2012). Mitochondrial molecular basis of sevoflurane and propofol cardioprotection in patients undergoing aortic valve replacement with cardiopulmonary bypass. Cellular Physiol Biochem.

[20] Uhlig C, Bluth T, Schwarz K, Deckert S, Heinrich L, De Hert S, Landoni G, Serpa Neto A, Schultz MJ, Pelosi P (2016). Effects of volatile anesthetics on mortality and postoperative pulmonary and other complications in patients undergoing surgery: a systematic review and meta-analysis. Anesthesiology.

[21] Grossgasteiger M, Hien MD, Graser B, Rauch H, Gondan M, Motsch J, Rosendal C (2013). Assessment of left ventricular size and function during cardiac surgery. An intraoperative evaluation of six two-dimensional echocardiographic methods with real time three-dimensional echocardiography as a reference. Echocardiography.

[22] Stanton T, Marwick TH (2010). Assessment of subendocardial structure and function. J Am Coll Cardiol Img.

[23] Iwahashi N, Nakatani S, Kanzaki H, Hasegawa T, Abe H, Kitakaze M (2006). Acute improvement in myocardial function assessed by myocardial strain and strain rate after aortic valve replacement for aortic stenosis. J Am Soc Echocardiogr.

[24] Duncan AE, Kateby Kashy B, Sarwar S, Singh A, Stenina-Adognravi O, Christoffersen S, Alfirevic A, Sale S, Yang D, Thomas JD (2015). Hyperinsulinemic Normoglycemia does not meaningfully improve myocardial performance during cardiac surgery: a randomized trial. Anesthesiology.

[25] Howell NJ, Ashrafian H, Drury NE, Ranasinghe AM, Contractor H, Isackson H, Calvert M, Williams LK, Freemantle N, Quinn DW (2011). Glucose-insulin-potassium reduces the incidence of low cardiac output episodes after aortic valve replacement for aortic stenosis in patients with left ventricular hypertrophy: results from the hypertrophy, insulin, glucose, and electrolytes (HINGE) trial. Circulation.

[26] Shao D, Tian R (2015). Glucose transporters in cardiac metabolism and hypertrophy. Comprehensive Physiology.

[27] Ranasinghe AM, McCabe CJ, Quinn DW, James SR, Pagano D, Franklyn JA, Bonser RS (2006). How does glucose insulin potassium improve hemodynamic performance? Evidence for altered expression of beta-adrenoreceptor and calcium handling genes. Circulation.

[28] McNulty PH, Pfau S, Deckelbaum LI (2000). Effect of plasma insulin level on myocardial blood flow and its mechanism of action. Am J Cardiol.

[29] Duncan AE, Sarwar S, Kateby Kashy B, Sonny A, Sale S, Alfirevic A, Yang D, Thomas JD, Gillinov M, Sessler DI (2017). Early left and right ventricular response to aortic valve replacement. Anesth Analg.

[30] Maslow AD, Regan MM, Schwartz C, Bert A, Singh A (2004). Inotropes improve right heart function in patients undergoing aortic valve replacement for aortic stenosis. Anesth Analg.

[31] Lamb HJ, Beyerbacht HP, de Roos A, van der Laarse A, Vliegen HW, Leujes F, Bax JJ, van der Wall EE (2002). Left ventricular remodeling early after aortic valve replacement: differential effects on diastolic function in aortic valve stenosis and aortic regurgitation. J Am Coll Cardiol.

